# Supplementary material for: On the robustness of democratic electoral processes to computational propaganda
Source: Sci Rep. 2024 Jan 2;14:193. doi: 10.1038/s41598-023-50648-6 (PMC10762203; doi:10.1038/s41598-023-50648-6)
Supplement: Supplementary file 1 — Supplementary Information. [file 41598_2023_50648_MOESM1_ESM.pdf]

Supplementary Information for
On the Robustness of Democratic Electoral Processes to Computational Propaganda

Glory M. Givi, Robin Delabays, Matthieu Jacquemet, and Philippe Jacquod

I. OPINION VOLUME BALANCING

For a system with p parties, by definition [Eq. (1)], the opinion space is a $(p-1)$-dimensional subset of $\mathbb{R}^p$. Therefore, for simplicity's sake, we embed the regular $(p-1)$-simplex $S_{\rm reg}$ in $\mathbb{R}^{p-1}$.

Let $\boldsymbol{v}_1, ..., \boldsymbol{v}_p \in \mathbb{R}^{p-1}$ be p points and let

$$S \coloneqq \mathrm{conv}(\boldsymbol{v}_1, ..., \boldsymbol{v}_p)\,, \tag{S1}$$

be the $(p-1)$-*simplex* $S \subset \mathbb{R}^{p-1}$ with *vertices* $\boldsymbol{v}_1$, ..., $\boldsymbol{v}_p$. The convex hull of $p-1$ vertices of S is a $(p-2)$-simplex, called a *facet* of S. The facet of S not containing the vertex $\boldsymbol{v}_i$ is denoted by F_i (it is the facet opposite to $\boldsymbol{v}_i$ in S), that is

$$F_i \coloneqq \mathrm{conv}(\boldsymbol{v}_1, ..., \boldsymbol{v}_{i-1}, \boldsymbol{v}_{i+1}, ..., \boldsymbol{v}_p)\,, \qquad i \in \{1, ..., p\}\,. \tag{S2}$$

The angle between the facets F_i and F_j is called the *dihedral angle* $\alpha_{ij} \in [0, \pi]$ of S.

The simplex $S \subset \mathbb{R}^{p-1}$ is said to be *regular* if $\alpha_{ij} = \alpha_{kl} =: \alpha_{\rm reg}$ for all $1 \leq i, j, k, l \leq p$. It can be shown that $\alpha_{\rm reg} = \arccos\frac{1}{p-1}$ [1, Section 7.9]. For $1 \leq q \leq p$, a $(q-1)$-simplex $S \subset \mathbb{R}^{p-1}$ is said to be a $(q-1)$-*orthoscheme* if $\alpha_{ij} = \frac{\pi}{2}$ if $|i-j| > 1$. For instance, a 2-orthoscheme is a right triangle, and the facets of a $(q-1)$-orthoscheme are $(q-2)$-orthoschemes.

The regular $(p-1)$-simplex $S_{\rm reg}$ can be dissected into $p!$ isometric $(p-1)$-orthoschemes by means of the recursive *barycentric decomposition* as follows (see Fig. S1). For $1 \leq k \leq p$, denote by $\boldsymbol{v}_{1,...,k}$ the barycenter of the convex hull $\mathrm{conv}(\boldsymbol{v}_1, ..., \boldsymbol{v}_k)$. Because $S_{\rm reg}$ is regular (so that all its d-dimensional faces are regular d-simplices themselves, $0 \leq d \leq p-1$), $\boldsymbol{v}_{1,...,k}$ is the center of the $(k-1)$-sphere inscribed in the convex hull of $\boldsymbol{v}_1, ..., \boldsymbol{v}_k$ (and of the $(k-1)$-sphere circumscribed around the convex hull of $\boldsymbol{v}_1, ..., \boldsymbol{v}_k$, etc.). For instance, $\boldsymbol{v}_{1,2}$ is the midpoint of the edge $[\boldsymbol{v}_1, \boldsymbol{v}_2]$ of $S_{\rm reg}$, $\boldsymbol{v}_{1,2,3}$ is the center of the regular triangle with

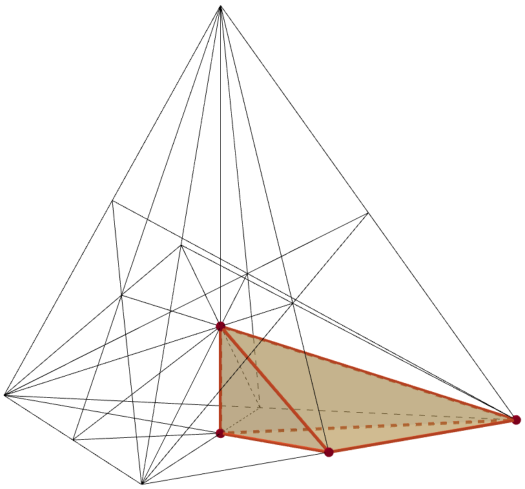

Figure S1. The regular 3-simplex S_{reg} and one of its 24 isometric fundamental orthoschemes O. Notice that here $p = 4$.

vertices $\boldsymbol{v}_1, \boldsymbol{v}_2, \boldsymbol{v}_3$ (a 2-dimensional face of S_{reg}), and $\boldsymbol{v}_{1,\dots,p}$ is the center of S_{reg}. Then, the convex hull

$$O_{1,\dots p} \coloneqq \mathrm{conv}(\boldsymbol{v}_1, \dots, \boldsymbol{v}_{1,\dots,p}) \,, \tag{S3}$$

is a $(p-1)$-orthoscheme whose non-right dihedral angles are $\frac{\pi}{3}, \dots, \frac{\pi}{3}, \frac{\alpha_{\mathrm{reg}}}{2}$.

The procedure described above can be performed for any permutation of $\{1, \dots, p\}$. Let $\mathcal{S}_p$ denote the group of all permutations of p objects, and let $(i_1, \dots, i_p) \in \mathcal{S}_p$ be such a permutation. Then, the corresponding orthoscheme $O_{i_1,\dots,i_p}$ is given by

$$O_{i_1,\dots,i_p} = \mathrm{conv}(\boldsymbol{v}_{i_1}, \boldsymbol{v}_{i_1,i_2}, \dots, \boldsymbol{v}_{i_1,\dots,i_p}) \,. \tag{S4}$$

Because S_{reg} is regular, $O_{i_1,\dots,i_p}$ is isometric to $O_{1,\dots,p}$ for any permutation $(i_1, \dots, i_p) \in \mathcal{S}_p$. This leads to a natural bijection between the set of isometric $(p-1)$-orthoschemes whose union is S_{reg} and the set $\mathcal{S}_p$.

Without loss of generality, let us consider the orthoscheme $O \coloneqq O_{1,\dots,p}$. Denote by $F_{1,\dots,k}$ the facet of O opposite to $v_{1,\dots,k}$. Now move the vertex $\boldsymbol{v}_1$ along the edge $[\boldsymbol{v}_1, \boldsymbol{v}_{1,\dots,p}]$ towards the vertex $\boldsymbol{v}_{1,\dots,p}$, to a point $\boldsymbol{s} \in [\boldsymbol{v}_1, \boldsymbol{v}_{1,\dots,p}]$. Let T be the simplex given by

$$T \coloneqq \mathrm{conv}(\boldsymbol{s}, \boldsymbol{v}_{1,2}, \dots, \boldsymbol{v}_{1,\dots,n+1}) \,. \tag{S5}$$

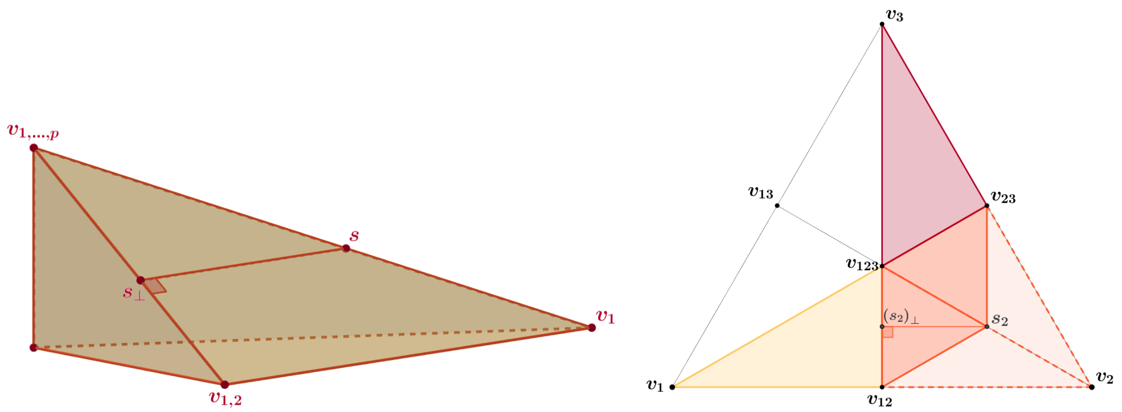

Figure S2. Left: Vertex $\boldsymbol{s}$ in the segment $[\boldsymbol{v}_1, \boldsymbol{v}_{1,\dots,p}]$ and its projection on the facet F_1. Right: Explicit realization of the construction of the left panel for $p = 3$.

For a given proportionality coefficient $\kappa \in [0, 1]$, we want to know where to set $\boldsymbol{s} = \boldsymbol{s}(\kappa)$ on $[\boldsymbol{v}_1, \boldsymbol{v}_{1,\dots,p}]$ so that

$$\mathrm{vol}_{p-1}(T) = \kappa \cdot \mathrm{vol}_{p-1}(O)\,. \tag{S6}$$

It is clear that $\kappa = 0 \Leftrightarrow \boldsymbol{s} = \boldsymbol{v}_{1,\dots,p}$ and that $\kappa = 1 \Leftrightarrow \boldsymbol{s} = \boldsymbol{v}_1$.

Denote by $\boldsymbol{s}_\perp$ the orthogonal projection of $\boldsymbol{s}$ on F_1, that is, $\boldsymbol{s}_\perp \in F_1$ and $[\boldsymbol{s}, \boldsymbol{s}_\perp] \perp F_1$. The edge $[\boldsymbol{v}_1, \boldsymbol{v}_{1,2}]$ of O is orthogonal to F_1, since

$$[\boldsymbol{v}_1, \boldsymbol{v}_{1,2}] = \bigcap_{k=3}^{p} F_{1,2,\dots,k}\,, \tag{S7}$$

and since $F_{1,\dots,k}$ is orthogonal to F_1 for $3 \le k \le p$ (because O is an orthoscheme). See Fig. S2 for an illustration of these observations.

Let F_s be the facet of T opposite to $\boldsymbol{s}$. Because the segments $[\boldsymbol{v}_1, \boldsymbol{v}_{1,2}]$ and $[\boldsymbol{s}, \boldsymbol{s}_\perp]$ are orthogonal to F_1 and F_s respectively, the volumes of O and T are given by

$$\mathrm{vol}_{p-1}(O) = \frac{1}{p-1} \cdot \mathrm{dist}(\boldsymbol{v}_1, \boldsymbol{v}_{1,2}) \cdot \mathrm{vol}_{p-2}(F_1)\,, \tag{S8}$$

and

$$\mathrm{vol}_{p-1}(T) = \frac{1}{p-1} \cdot \mathrm{dist}(\boldsymbol{s}, \boldsymbol{s}_\perp) \cdot \mathrm{vol}_{p-2}(F_s)\,. \tag{S9}$$

Now observe that

$$F_1 = \mathrm{conv}(\boldsymbol{v}_{1,2}, \dots, \boldsymbol{v}_{1,\dots,p}) = F_s\,, \tag{S10}$$

so that the quotient of the volumes of O and T is given by

$$\frac{\mathrm{vol}_{p-1}(T)}{\mathrm{vol}_{p-1}(O)} = \frac{\mathrm{dist}(\boldsymbol{s}, \boldsymbol{s}_\perp)}{\mathrm{dist}(\boldsymbol{v}_1, \boldsymbol{v}_{1,2})}, \tag{S11}$$

so that Condition (S6) is equivalent to

$$\mathrm{dist}(\boldsymbol{s}, \boldsymbol{s}_\perp) = \kappa \cdot \mathrm{dist}(\boldsymbol{v}_1, \boldsymbol{v}_{1,2}). \tag{S12}$$

Let us now investigate further the relative positions of the segments $[\boldsymbol{v}_1, \boldsymbol{v}_{1,2}]$ and $[\boldsymbol{s}, \boldsymbol{s}_\perp]$. Since $\dim(F_1) = p-2$ (because F_1 is the convex hull of $p-1$ vertices) and since $[\boldsymbol{v}_1, \boldsymbol{v}_{1,2}] \perp F_1$ (as stated above), it follows that the segments $[\boldsymbol{s}, \boldsymbol{s}_\perp]$ and $[\boldsymbol{v}_1, \boldsymbol{v}_{1,2}]$ are parallel. Moreover, by construction, the points $\boldsymbol{v}_1$, $\boldsymbol{v}_{1,2}$ and $\boldsymbol{s}$ all belong to the 2-face

$$\triangle := \mathrm{conv}(\boldsymbol{v}_1, \boldsymbol{v}_{1,2}, \boldsymbol{v}_{1,\ldots,p}), \tag{S13}$$

of O. Hence, because $[\boldsymbol{s}, \boldsymbol{s}_\perp] \,//\, [\boldsymbol{v}_1, \boldsymbol{v}_{1,2}]$, it follows that $\boldsymbol{s}_\perp$ is contained in $\triangle$ as well. This in turn implies that the point $\boldsymbol{s}_\perp$ is on the segment $[\boldsymbol{v}_{1,2}, \boldsymbol{v}_{1,\ldots,p}]$, since it is an edge of F_1. Let

$$f := \mathrm{conv}(\boldsymbol{s}, \boldsymbol{s}_\perp, \boldsymbol{v}_{1,\ldots,p}), \tag{S14}$$

denote the 2-face of T with vertices $\boldsymbol{s}$, $\boldsymbol{s}_\perp$ and $\boldsymbol{v}_{1,\ldots,p}$. From the discussion above we deduce that $f \subset \triangle$, and that both are right-angled triangles (f at $\boldsymbol{s}_\perp$, and $\triangle$ at $\boldsymbol{v}_{1,2}$) sharing the vertex $\boldsymbol{v}_{1,\ldots,p}$.

These observations allow us to deduce that Condition (S12) is equivalent to

$$\mathrm{dist}(\boldsymbol{s}, \boldsymbol{v}_{1,\ldots,p}) = \kappa \cdot \mathrm{dist}(\boldsymbol{v}_1, \boldsymbol{v}_{1,\ldots,p}), \tag{S15}$$

leading us to the *a priori* somewhat intuitive (or seemingly too nice to be true) fact:

Proposition 1. *In order to reduce the volume of O to a proportion $\kappa \in [0,1]$, the vertex $\boldsymbol{v}_1$ has to be moved towards $\boldsymbol{v}_{1,\ldots,p}$ to the point $\boldsymbol{s} \in [\boldsymbol{v}_1, \boldsymbol{v}_{1,\ldots,p}]$ dividing the edge $[\boldsymbol{v}_1, \boldsymbol{v}_{1,\ldots,p}]$ under the same proportion κ.*

Now that we have a way to control the volume of an orthoscheme while keeping one of its facets intact, we turn to the main question of interest for the opinion generation problem.

Without loss of generality, label the parties from 1 to p to match the left-right political spectrum. In order for an opinion to be consistent, we suppose that party i is the most favored (for some $i \in \{1, \ldots, p\}$), and that the adhesion to party j is smaller than (respectively,

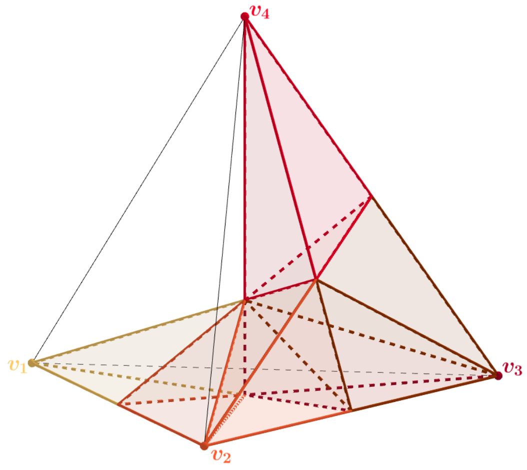

Figure S3. Illustration of the set C of admissible opinions for $p = 4$.

greater than) the adhesion to party $j+1$ if $j \in \{1, ..., i-1\}$ (respectively, $j \in \{i, ..., p\}$). These constraints reflect the assumption that as parties are further from the agent's favorite party on the political spectrum, the agent's adhesion towards them decreases. Let $\boldsymbol{x} \in S_{\text{reg}}$ be a point representing the opinion of the agent as follows. The agent favors party j over party k if and only if $\text{dist}(\boldsymbol{x}, \boldsymbol{v}_j) < \text{dist}(\boldsymbol{x}, \boldsymbol{v}_k)$. The set C of all points $\boldsymbol{x} \in S_{\text{reg}}$ representing admissible opinions (in the sense described above) is given by the non-convex polyhedron (see Fig. S3)

$$C = \left\{ \boldsymbol{x} \in S_{\text{reg}} \;\middle|\; \begin{array}{c} \text{There is an } i \in \{1, ..., n+1\} \text{ such that} \\ \text{dist}(\boldsymbol{x}, \boldsymbol{v}_j) \geq \text{dist}(\boldsymbol{x}, \boldsymbol{v}_{j+1}) \text{ for } j \in \{1, ..., i-1\} \text{ and} \\ \text{dist}(\boldsymbol{x}, \boldsymbol{v}_j) \leq \text{dist}(\boldsymbol{x}, \boldsymbol{v}_{j+1}) \text{ for } j \in \{i, ..., p-1\} \end{array} \right\}. \tag{S16}$$

Recall that the regular $(p-1)$-simplex S_{reg} can be decomposed into $p!$ isometric orthoschemes thanks to the barycentric decomposition. Each vertex $\boldsymbol{v}_i$ of S_{reg} is a vertex of $(p-1)!$ such orthoschemes. Let R_i be the union of the $(p-1)!$ orthoschemes having $\boldsymbol{v}_i$ as a vertex. Then $\boldsymbol{x} \in R_i$ if and only if party i is the agent's favorite party. Hence, a point $\boldsymbol{x} \in R_i$ represents a consistent opinion (in the sense described above) if and only if it satisfies the distance conditions from (S16) for that i. For instance, $C \cap R_1 = O$, the orthoscheme with vertices $\boldsymbol{v}_1, ..., \boldsymbol{v}_{1,...,p}$ described above.

Recall that the permutations in $\mathcal{S}_p$ are in 1-1 correspondence with the orthoschemes in the barycentric decomposition of S_{reg}, which in turns provides a 1-1 correspondence with all possible ordering of the parties: the permutation $(i_1, ..., i_p)$ corresponds to the orthoscheme

conv$(\boldsymbol{v}_{i_1}, \boldsymbol{v}_{i_1,i_2}, ..., \boldsymbol{v}_{i_1,...,i_p})$, which corresponds to the opinion with party i_1 being the favorite party, party i_2 the next favorite party (since $\boldsymbol{v}_{i_1,i_2}$ is the midpoint of the edge $[\boldsymbol{v}_{i_1}, \boldsymbol{v}_{i_2}]$), and party i_p the least favorite party.

Conversely, any party ordering corresponds to a permutation in $\mathcal{S}_p$. Therefore, an opinion is consistent (in the sense described above) if and only if its corresponding permutation $(i_1, ..., i_p)$ satisfies the following conditions:

$$i_k < i_l < i_1 \Rightarrow k > l\,, \qquad \text{and} \qquad i_k > i_l > i_1 \Rightarrow k > l\,. \tag{S17}$$

In other words, in the sequence of indices $i_1, ..., i_p$, the elements of the subsequences $i_1-1, ..., 1$ and $i_1+1, ..., p$ must appear precisely in that order, but elements from different subsequences can be permuted. There are $\binom{p-1}{i_1-1}$ such permutations starting with i_1. Hence, for all $i \in \{1, ..., p\}$, $C \cap R_i$ consists in the $\binom{p-1}{i-1}$ orthoschemes corresponding to consistent opinions. In particular, $C \cap R_1$ is the orthoscheme $O_{1,...,p}$ and $C \cap R_p$ is the orthoscheme $O_{p,...,1}$. It follows that the set C of points in S_{reg} corresponding to consistent opinions is a non-convex polyhedron, obtained as the union of

$$\sum_{i=1}^{p} \binom{p-1}{i-1} = 2^{p-1}\,, \tag{S18}$$

isometric orthoschemes described above. The vertices $\boldsymbol{v}_1, ..., \boldsymbol{v}_p$ form a subset of the vertices of C, and the vertex $\boldsymbol{v}_i$ is a vertex of $\binom{p-1}{i-1}$ such orthoschemes.

In order for the opinion generation process to be unbiased, the probability to generate an opinion representative $\boldsymbol{x} \in C \cap R_i$ should be the same for all $i \in \{1, ..., p\}$. The discussion above provides a natural way to generate such opinions. Observe that for each permutation $(i_1, ..., i_p)$, the point $\boldsymbol{v}_{i_1,...,i_p}$ is the center $\boldsymbol{c}$ of S_{reg}. For each vertex $\boldsymbol{v}_i$, move it along the ray $[\boldsymbol{v}_i, \boldsymbol{c}]$ towards $\boldsymbol{c}$ to the point $\boldsymbol{s}_i$ such that

$$\text{dist}(\boldsymbol{s}_i, \boldsymbol{c}) = \frac{1}{\binom{p-1}{i-1}} \cdot \text{dist}(\boldsymbol{v}_i, \boldsymbol{c})\,. \tag{S19}$$

Notice that this procedure preserves the existing interfaces between $C \cap R_i$ and $C \cap R_j$ for all $i \neq j \in \{1, ..., p\}$, so that the non-convex polyhedron C_{r} obtained that way can be seen a non-uniform radial retractation of C with respect of $\boldsymbol{c}$. The set C_{r} is the colored area in the right panel of Fig. S2.

Because of Proposition 1, the volumes of all orthoschemes building $C \cap R_i$ are reduced by a factor $\binom{p-1}{i-1}$, so that each of the resulting regions $C_{\text{r}} \cap R_i$ has the same volume, which

is equal to the volume of the isometric orthoschemes $O_{1,...,p}$ and $O_{p,...,1}$ (one has indeed that $C \cap R_1 = C_{\mathrm{r}} \cap R_1$ and $C \cap R_p = C_{\mathrm{r}} \cap R_p$).

We summarize the process for generating a random opinion, drawn uniformly in the admissible opinion space:

1. Take the barycentric decomposition of the standard regular simplex S_{reg} into $p!$ isometric orthoschemes.

2. From these orthoschemes, only consider the 2^{p-1} ones corresponding to consistent opinions (their union is the polyhedron C).

3. For all $i \in \{1, ..., p\}$, retract the orthoschemes in C containing the vertex $\boldsymbol{v}_i$ (their union is the region R_i) radially with respect to the center $\boldsymbol{c}$ of S_{reg} from a factor $\binom{p-1}{i-1}$. The union of these retracted orthoschemes forms the polyhedron C_{r}. As a result, $\mathrm{vol}_{p-1}(C_{\mathrm{r}} \cap R_i) = \mathrm{vol}_{p-1}(C_{\mathrm{r}} \cap R_j)$ for all $i \neq j \in \{1, ..., p\}$.

4. In C_{r}, randomly pick N points, where N is the desired amount of opinions to be generated. The probability that a point $x \in C_{\mathrm{r}}$ belongs to the region $C_{\mathrm{r}} \cap R_i$ is the same for all $i \in \{1, ..., p\}$.

An implementation of this procedure is available online [2].

II. SPECTRUM OF THE DYNAMICS MATRIX

Lemma. *The matrix $M := D^{-1}L + I_n$ [as defined in the Methods Section, Eq. (5)] has a real positive spectrum.*

Proof. First, let us identify the eigenvalues of $D^{-1}L$, which are the zeros of the characteristic polynomial

$$\begin{aligned} 0 &= \det\left(\lambda I_n - D^{-1}L\right) \\ &= \det\left[D^{-1/2}\left(\lambda I_n - D^{-1/2}LD^{-1/2}\right)D^{1/2}\right] \\ &= \det\left(\lambda I_n - D^{-1/2}LD^{-1/2}\right) . \end{aligned} \tag{S20}$$

We then see that the spectrum of $D^{-1}L$ coincide with the spectrum of $D^{-1/2}LD^{-1/2}$. The matrix $D^{-1/2}LD^{-1/2}$ being real symmetric, its spectrum (and consequently the spectrum of

$D^{-1}L$) is real. Furthermore, by definition of M, λ is an eigenvalue of $D^{-1}L$ if and only if $\lambda + 1$ is an eigenvalue of M. The spectrum of M is then real.

Second, by Gershgorin's Circles Theorem [3], the eigenvalues of M have a positive real part, which concludes the proof. □

III. TARGETING ELECTORAL UNITS

When influencing agents in order to change the election outcome in an electoral unit, one would intuitively target the agents close to the center of the opinion distribution in priority as their vote is likely to be easier to change. This line of reasoning is confirmed in the left panel of Fig. S4, where we compare the effort needed to change the outcome of a vote by influencing the agents closer to the barycenter of the opinion space $\xi_{\rm min}$ to the effort when target agents at random $\xi_{\rm rand}$.

When a country is composed of multiple electoral units, there are two natural ways to select which electoral unit to target in priority. One can either target agents in the electoral unit with the lowest population, because changing the outcome in such a unit requires to influence less agents, or one can target the electoral unit with smallest relative majority, because such a unit is close to a change of outcome already. In the right panel of Fig. S4, we show that targeting the electoral units with lower relative majority is generally more efficient than targeting the electoral units with lower population. Our strategy to overturn elections is therefore to target weakly opiniated agents in electoral units with lower population.

IV. MODEL VALIDATION AND THE US HOUSE OF REPRESENTATIVES ELECTION

For the validation of our model, we compare historical and numerical volatility of the House of Representatives elections in the US, between 2012 and 2020 [4].

We measure the historical volatility $V_{\rm h}$ of the House of Representatives election in each district by counting the number of times the majority (Democrats or Republicans) has changed from 2012 to 2020. A single representative is elected at the level of the districts for the House of Representatives elections. The more this number changes over time, the more volatile the electoral unit is. This volatility measure is illustrated in Fig. 1a. Over the

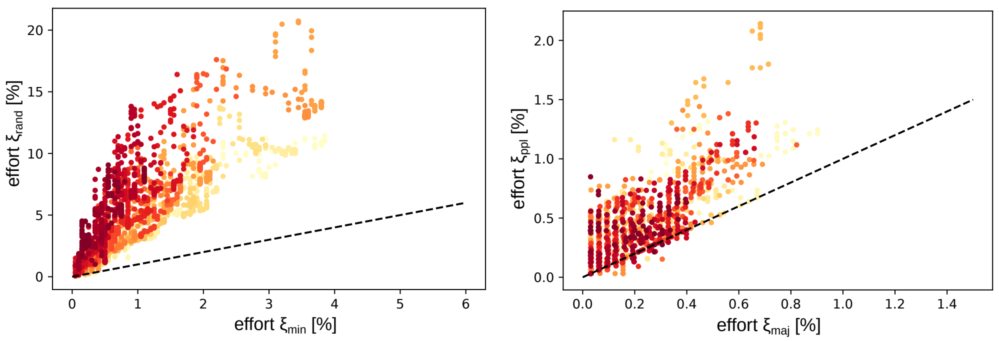

Figure S4. Left: The effort needed to change the outcome of election by targeting the agents close to the barycenter, ξ_{min}, versus targeting random agents, ξ_{rand}. Each point denotes a natural opinion with different confidence bound ϵ, polarization and bias. Right: The effort needed to change the outcome of election by targeting the electoral units with the minimal majority, ξ_{maj}, and the electoral units with the minimal population, ξ_{ppl}, with 7 states. We consider each state to have $400-500$ agents. In both panels, different colors indicate different values of confidence bound ϵ, polarization, and bias.

period $2012-2020$, 78 districts changed majority at least once (Fig. 1a in the main text).

To compute the numerical volatility V_{t} given by our model, we attribute an *effort budget* $\Xi \in [0.0025, 0.2]$ to influence a certain percentage of agents in the country, which is distributed evenly among the electoral units, i.e., each electoral unit, we can influence up to a fraction Ξ of the agents. We then simulate the election in each district with $n_{\mathrm{d}} = 501$ agents each and compute the number of times this influence can change the outcome in each of these electoral units over 100 realisations of natural opinions for each type of distribution described in Sec. C. After influence, each district may or may not change the majority. Averaging overall realisations gives our estimate of volatility.

We calculate the Pearson correlation coefficient between the numerically computed volatility and the historical volatility from the 2012 to 2020 US House of Representatives elections.

V. ROBUSTNESS OF BIPARTITE SYSTEMS IN SINGLE ELECTORAL UNITS

In the main text we mentioned that biases parameters μ and ρ of Eq. (12) result in clear-cut electoral results, i.e. where there is a substantial percentage difference between

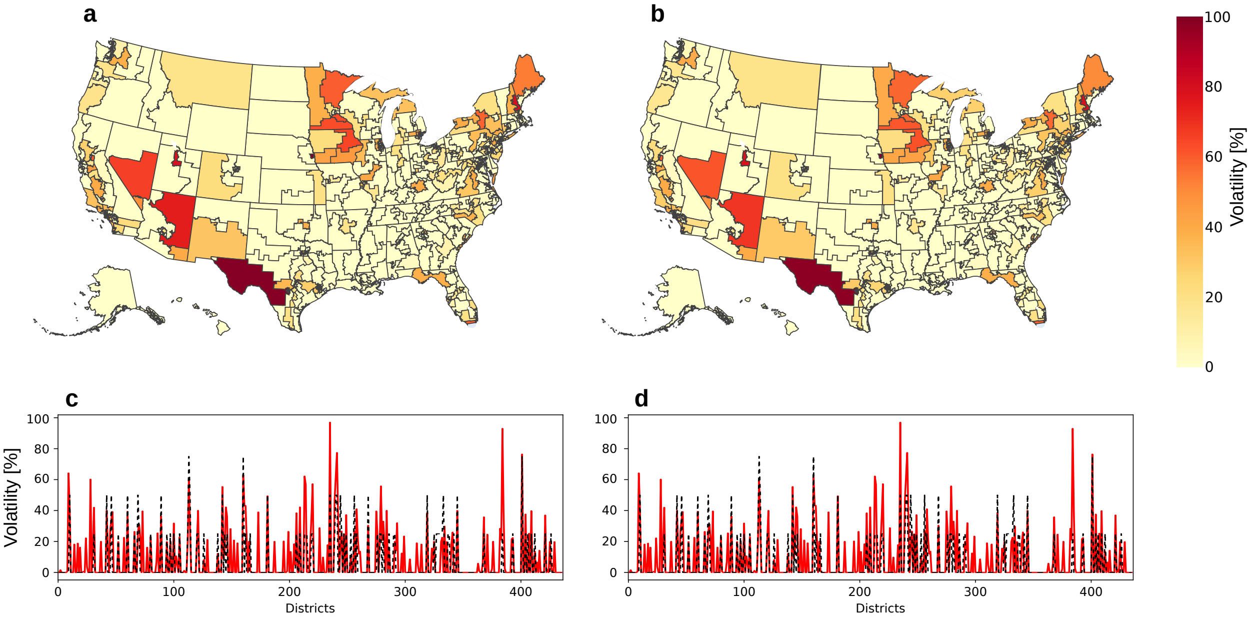

Figure S5. **Supplementary figure:** Color scale of the simulated volatility **a** for a bigaussian distribution with bias and **b** for a gaussian distribution with bias. Panel **c** (resp. **d**) shows the line plot of the above color scale in panel **a** (resp. **b**) where districts are arranged along the horizontal axis. The dashed black line shows the historic volatility and the plain red line shows the simulated volatility.

the winner and the first runner-up. It is then expected that such a result is robust against opinion manipulation, because the opinion of a large numbers of voters need to be changed in that case. Fig. S6 confirms this expectation – increasing the electoral bias. with either μ or ρ increases the effort needed to change the electoral outcome. As mentioned in the main text, one concludes, rather trivially, that population with electoral biases are robust against computational propaganda.

VI. GERRYMANDERING

Geographical redistricting may change the outcome of an election in favor of one or another party in a state with several districts. Focusing on bipartite elections, the goal is to achieve a slight majority in favor of one party in as many districts as possible, while concentrating the votes of the other party in as few districts as possible. This procedure is called gerrymyndering and we briefly consider its impact on electoral robustness in bipartite

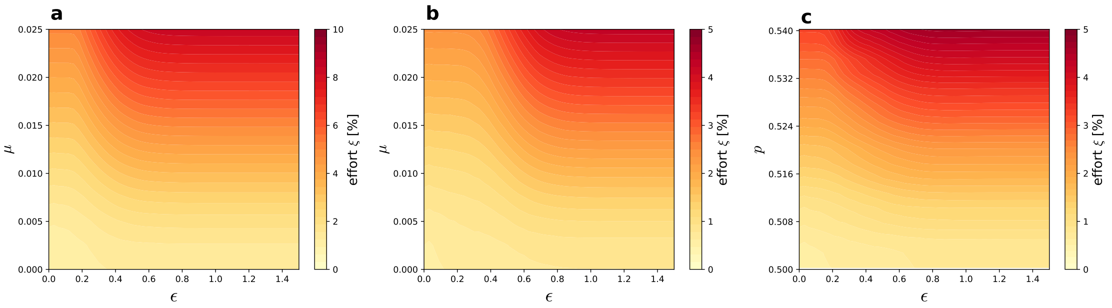

Figure S6. **Supplementary figure:** Average effort needed to change the outcome of an election with respect to different parameters of the opinion distributions of Eq. (12) and the confidence bound ϵ. The average is taken over 500 realisations of *natural opinion* with 2001 agents in an electoral unit for each simulation. **a** Average effort as a function of the confidence bound ϵ and the distribution bias μ, for a gaussian distribution ($\Delta = 0.0$, $\sigma = 0.2$, and $\rho = 0.5$). **b** Average effort as a function of the confidence bound ϵ and the distribution bias μ, for a bigaussian distribution ($\Delta = 0.5$, $\sigma = 0.2$, and $\rho = 0.5$). **c** Average effort as a function of the confidence bound ϵ and the balance parameter ρ, for a bigaussian distribution ($\Delta = 0.5$, $\sigma = 0.2$, and $\mu = 0.0$).

elections.

In Fig. S7 we show that gerrymandered bipartite elections are more robust than those where electorates have homogeneously distributed natural opinions, equally distributed between the two parties. This result is easily understood. Gerrymandering introduces an electoral bias in each district, therefore electoral outcomes have larger voting margins in favor of the winning party in each district. Many more voter opinions need to be reversed to change such electoral outcomes.

VII. ROBUSTNESS OF THE HOUSE OF REPRESENTATIVES ELECTIONS

In the main text (Fig. 3), we show how robustness evolves with respect to the opinion distance ϵ in randomly generated synthetic countries. Here we corroborate our findings on the US House of Representative elections between 2012 and 2020. We use the results of each of these elections as bias or shift in the opinion distribution. The simulations show little difference with the fully synthetic countries, except for the WTA system that appears to never be the most robust in this case (see Fig. S8).

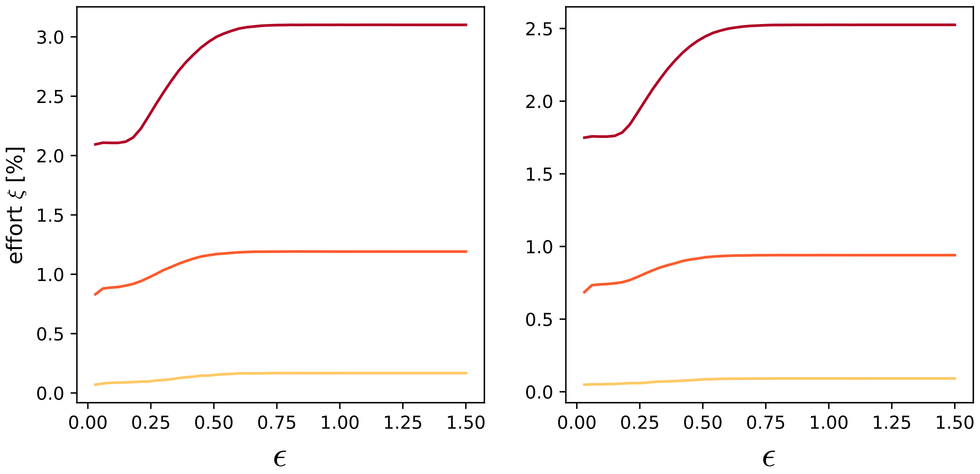

Figure S7. **Supplementary figure:** Average effort needed to change the outcome of a bipartite election in a state with five (left) and nine (right) electoral districts and for opinion distributions given in Eq. (12) with $\sigma = 0.2$, $\mu = 0$, $\rho = 0$ and $\Delta = 0$. Different levels of gerrymandering are considered: no gerrymandering where natural opinions are distributed equally between the two parties in each district (yellow curve), where redistricting leads to 52 % vs 48 % of natural opinions in all but one districts and 42 % vs. 58 % in the last one (orange), and to 55 % vs. 45 % in all but one districts and 30 % vs 70 % in the last one (red).

VIII. ROBUSTNESS FOR MULTIPARTITE SYSTEMS

In the main text (Figs. 4 and 5), we show the robustness of the election outcome in a single electoral unit with 6 parties. Our finding is that the average effort needed to change an election is dominated by the effort needed to make the extremist parties win (when they are the first runner). A direct consequence if this observation is that, as soon as the extremist parties do not reach the second rank of the elections (which eventually happens when increasing the opinion distance), the robustness of the system drastically falls.

We show in Figs. S9 and S10 these observations are valid a number of parties ranging from 3 to 7, without noticeable differences.

[1] H. S. M. Coxeter, *Regular polytopes*, 3rd ed. (Dover Publications, New York, 1973).

[2] Codes are attached to the submission. (2023).

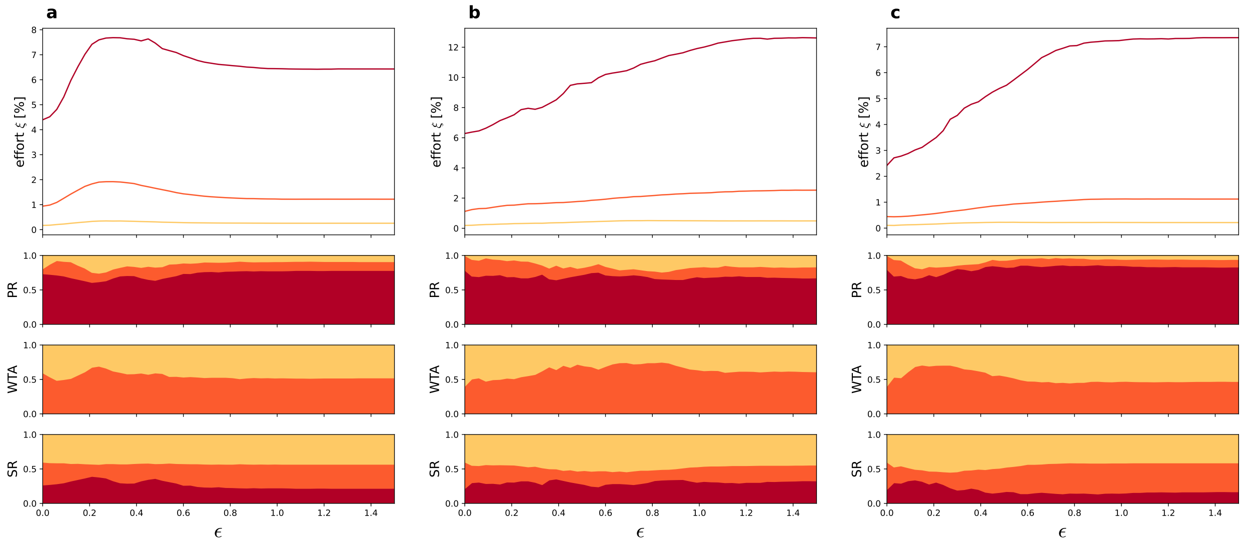

Figure S8. **Supplementary figure:** Robustness evaluation on a simulation of the US House of Representatives elections, between 2012 and 2020 (100 realization for each election). Elections are performed at the level of states, with results aggregated from the districts [4]. Top row: average effort needed to change the election outcome for three electoral systems (PR: red, WTA: orange, and SR: yellow) and three distributions of natural opinions. Bottom rows: Proportion of times each electoral system was the most robust (red), second most robust (orange), and least robust (yellow).

[3] R. A. Horn and C. R. Johnson, *Matrix Analysis* (Cambridge University Press, New York, 1994).

[4] "US federal election commission – election and voting information – election results," `https://www.fec.gov/introduction-campaign-finance/election-and-voting-information/`.

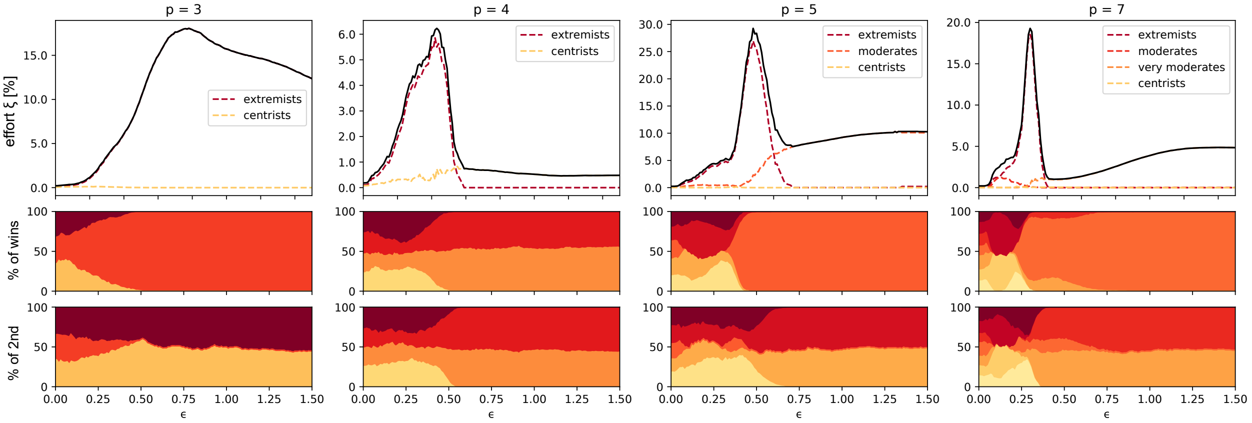

Figure S9. **Supplementary figure:** Same as Fig. 4 in the main text, for 3, 4, 5, and 7 parties (from left to right). Top row: average effort to change the election outcome, as a function of the confidence bound ϵ. The effort is broken down into the contribution of different realizations aggregated according to the position of the first runner-up in the political spectrum. Middle row: Proportion of times each party wins the elections. Bottom row: Proportion of times each party is the first runner.

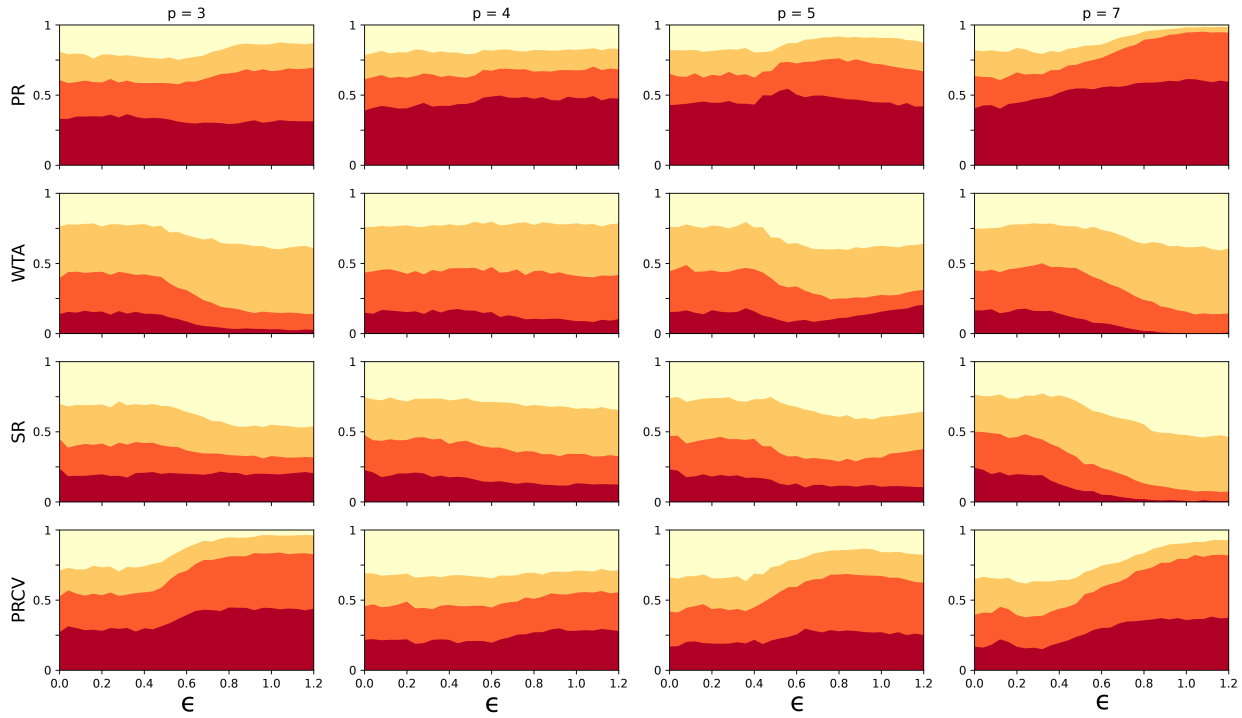

Figure S10. **Supplementary figure:** Same as Fig. 5 in the main text, for 3, 4, 5, and 7 parties (from left to right) and for the four electoral systems: PR, WTA, SR, and PRCV (from top to bottom). Proportion of realizations where the electoral system is the most robust (dark red), second most robust (orange), and least robust (yellow) in our simuliations.
